# Medically Tailored Meals: A Case for Federal Policy Action

**DOI:** 10.3390/healthcare13222899

**Published:** 2025-11-13

**Authors:** Catherine Macpherson, William H. Frist, Emily Gillen

**Affiliations:** 1 Mom’s Meals, Ankeny, IA 50021, USA; 2 Department of Cardiac Surgery, Vanderbilt University, Nashville, TN 37235, USA; 3 Avalere, Washington, DC 20005, USA

**Keywords:** medically tailored meals, health care cost reduction, nutrition security, chronic disease management, value-based care, health equity, medicare policy reform

## Abstract

**Background:** Poor nutrition drives chronic disease, health disparities, and rising health care costs in the United States. Medically tailored meals (MTMs), designed by registered dietitians, are a Food-as-Medicine intervention with potential to improve outcomes and reduce costs. This review synthesizes evidence on the clinical, economic, and policy implications of MTMs. **Methods:** We conducted a narrative review of peer-reviewed studies, real-world program evaluations, and policy analyses. Sources included PubMed, Google Scholar, and grey literature from government, nonprofit, and industry organizations. Articles and reports were included if they examined MTMs in Medicare, Medicaid, or other high-risk populations. **Results:** Evidence demonstrates that MTMs improve health outcomes, reduce hospitalizations, and lower total cost of care. Case studies from Medicaid and Medicare Advantage plans, including those administered by Mom’s Meals^®^, report reductions in emergency department visits, hospital readmissions, and total cost of care, alongside sustained high member satisfaction. Despite these findings, gaps in coverage and limited stakeholder awareness hinder broader access and adoption. **Conclusions:** Federal policy action can expand MTM availability and maximize utilization of existing benefits. Opportunities include establishing a Medicare Fee-for-Service demonstration, expanding and encouraging use in Medicare Advantage, and leveraging MTMs within Center for Medicare and Medicaid Innovation models. Broader implementation and utilization could reduce the nation’s chronic disease burden, advance health equity, and promote value-based care.

## 1. Background

**Figure 1:**
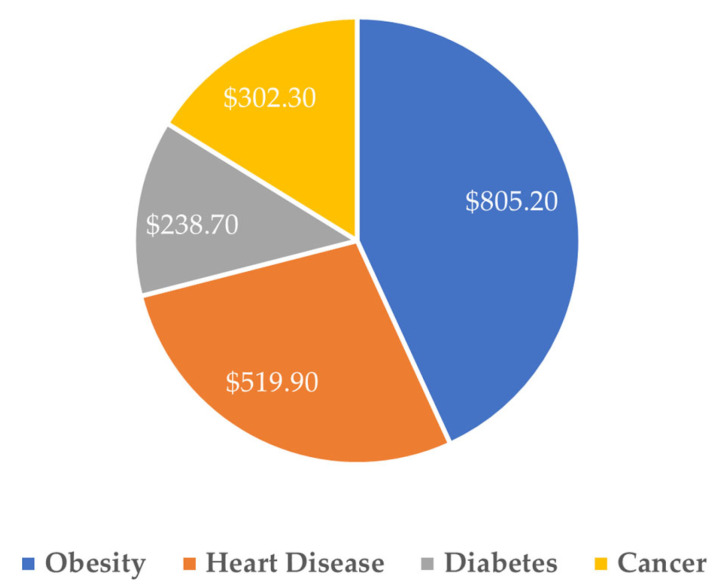
Total cost due to treatment and lost productivity, nutrition-impacted chronic conditions, 2020 (billions) [2]. Reproduced with permission from American Action Forum, The Economic Costs of Poor Nutrition; published by American Action Forum, 2022 [2].

The role of poor nutrition in driving higher health care costs and poor outcomes has moved beyond debate. Inadequate nutrition has a multiplying effect in driving not only the costliest health care conditions and comorbidities but also the mortality rates associated with them, contributing to the nation’s leading causes of death, including heart disease (680,981 deaths), cancer (613,352), stroke (162,639), and diabetes (95,190) annually [1]. The American Action Forum recently quantified the estimated total cost due to treatment and lost productivity of four nutrition-impacted chronic conditions, finding that annual costs in 2020 totaled more than $1.8 trillion dollars as shown in Figure 1 [2].

Medicare-aged beneficiaries are among the most impacted populations in terms of nutrition-responsive issues, and it is estimated that 95% of older adults manage at least one chronic health care condition [3], while rates of adherence to dietary guidelines related to these conditions remain low. More than 50% of older adults are malnourished or at risk of malnutrition, which is associated with poorer health outcomes [4].

Food-insecure older adults, aged 60 and over, are 53% more likely to suffer a heart attack, 40% more likely to suffer from congestive heart failure, and 52% more likely to develop asthma [5]. From a cost perspective, food-insecure adults with chronic diseases have higher health care costs. One study found the incremental costs associated with food insecurity among older adults with a chronic condition ranged from $530 (cancer) to $1740 (arthritis) per person per year (2015 USD) [6]. The scale of the nutrition burden on Medicare-aged patients is clear. Together, poor nutrition and nutrition insecurity are meaningful drivers of higher health care cost and poor outcomes, with a disproportionate impact on government-sponsored insurance markets and historically marginalized communities [3,5].

While progress has been made in developing condition-specific nutrition interventions, a continued lack of access to and lack of utilization of these interventions threatens to both increase health care costs, estimated at $16 billion annually, including about $1800 in excess costs per affected adult, and hinder efforts to curtail rising rates of cardiovascular and metabolic diseases [2,4,6]. Medically tailored meals (MTMs), designed to address individual clinical needs, offer a cost-effective strategy to improve outcomes and reduce avoidable utilization. However, their integration into federal programs remains inconsistent and underutilized [7]. Our review is particularly timely because of the growing role of nutrition in chronic disease prevention, alongside renewed federal interest in Food-is-Medicine (FIM) models, which has accelerated efforts to integrate MTMs into health care policy and practice. A narrative review is needed to synthesize emerging clinical and economic findings and to translate them into actionable policy opportunities.

This narrative review synthesizes peer-reviewed literature and real-world evaluations to assess the clinical and economic impacts of MTMs. Considering the growing burden of nutrition-related chronic disease and ongoing budgetary pressures, we examine the potential of MTMs as a scalable, evidence-based solution. We also explore policy pathways, including Medicare Fee-for-Service (FFS) demonstrations, Medicare Advantage (MA) FIM benefit models and expanded use within Center for Medicare and Medicaid Innovation (CMMI) programs, that could increase access and impact.

## 2. Methods

This paper is a narrative review intended to synthesize the existing peer-reviewed evidence, real-world program evaluations, and relevant policy analyses on MTMs. The purpose of this review is to provide a comprehensive overview of the clinical, economic, and policy implications of MTMs and to identify policy solutions to leverage the clinical and cost advantages of MTMs.

To identify relevant literature, we conducted searches of PubMed and Google Scholar for English-language publications through July 2025, using unstructured combinations of the following terms: “medically tailored meals”, “food is medicine”, “nutrition interventions”, “Medicare”, “Medicaid”, “health care utilization”, and “cost savings”. Roughly 30 sources of information were examined to provide a broad overview of available evidence.

We included studies and reports that examined MTMs in U.S. populations with diet-sensitive chronic conditions, post-discharge care, or high-risk Medicare and Medicaid beneficiaries, and that reported on clinical, utilization, or economic outcomes. Non-tailored nutritional interventions (e.g., general meal delivery not linked to medical need) were excluded.

In addition to peer-reviewed studies, we incorporated grey literature such as government reports, nonprofit white papers, and health plan evaluations. These sources were included because they represent some of the only available data on specific MTM implementations and provide timely and practical insights for policy discussions. Given the narrative design, no formal protocol or quality assessment was undertaken. Instead, the emphasis was on breadth of coverage and synthesis of evidence to inform future policy and research directions.

## 3. Results

### 3.1. Cost Savings of Medically Tailored Meals

In addressing the gap in adequate nutrition, MTMs have become a common offering in Medicaid and MA plans. MTMs are differentiated from other forms of FIM in that they are designed by a registered dietitian (RD) in order to alleviate or prevent a medical event through the provision of customized meals or menus. For example, a patient with kidney disease may receive meals lower in phosphorus and potassium, while a cardiac patient may receive meals lower in sodium or certain fats.

The Bipartisan Policy Center has estimated that $1.57 is saved for every dollar invested in MTMs for Medicare beneficiaries with certain chronic conditions through reductions in hospital readmissions post-discharge [8]. In Medicaid, it is estimated that spending is $712 lower per month for enrollees with chronic diseases who received two MTMs per day, 5 days a week, for an average of 12 months. Savings are primarily due to reductions in inpatient hospital and skilled nursing facility stays [9].

Furthermore, recently completed health plan case studies which leverage data from Mom’s Meals^®^ MTM programs delivered in partnership with AmeriHealth Caritas District of Columbia and a Medicaid Plan in California and that are considered grey literature show that MTMs are a cost-effective method to reduce hospital readmissions, total cost of care, and emergency department (ED) utilization, reporting up to a $10 million total cost reduction and 20% fewer readmissions in post-discharge participants, all while maintaining high member satisfaction [10].

### 3.2. Clinical, Observational and Health Policy Modeling Evidence for Medically Tailored Meals

In addition to plan-specific case studies, evidence illustrating the impact of MTMs is diverse in terms of use cases and conditions treated. As summarized in Table 1 [9,11,12,13,14,15,16,17,18,19,20,21,22,23,24,25,26,27], recent clinical studies, health policy modeling, and health plan cohort analyses have quantified the health impact of MTM interventions. These analyses typically focus on MTMs used as part of either chronic condition treatment or following an acute event, with meaningful impact on overall spend, ED visits, inpatient hospital utilization, clinical status, and other measures of patient well-being.

These real-world case studies, modeled health policy studies, cohort analyses, and published clinical evidence illustrate the meaningful and specific impact that MTMs can have on a wide array of beneficiaries, and further the need for national policymaking to leverage the benefit of MTMs for a broader population.

### 3.3. Barriers to Medically Tailored Meals Access

Despite the established benefits of MTMs, particularly for Medicare and Medicaid beneficiaries, many barriers exist to widespread availability in the form of coverage gaps and awareness of FIM benefits. Addressing these barriers to access represents a meaningful opportunity to drive improved health outcomes for Americans.

Table 2 highlights the coverage barriers for MTMs in the context of Medicare FFS, MA, and state Medicaid plans [28]. Addressing these access gaps will require legislative or regulatory action on the part of policymakers and are likely to be dependent on a refined view of implementation and program design considerations.

In addition to a lack of coverage and access to MTM benefits, there remain meaningful gaps in the awareness of FIM and MTMs among key health care stakeholders. According to a recent national survey, knowledge of specific MTM offerings and coverage remains below 30% [32]. Yet, after learning what MTMs are, more than half of respondents expressed interest in participating, indicating a significant mismatch between knowledge and demand. This imbalance highlights a nearly two-to-one gap between the public’s enthusiasm for MTM participation and their baseline awareness of such programs.

These barriers represent meaningful challenges related to the scalability of MTM and FIM benefits, which will need to be addressed with specific legislative and regulatory solutions, as well as opportunities, which could yield innovative solutions from within or for the health care industry. Progress toward addressing these barriers and solutions will be necessary to ensure population-wide nutrition improvement.

## 4. Discussion

Based on this review of literature, there is both clinical and economic promise of MTMs across a range of populations and care settings. But despite these consistent findings, the benefits of MTMs remain unevenly realized due to coverage gaps, inconsistent implementation, and limited awareness among patients, providers, and policymakers. Specifically, we outline policy pathways that can translate the evidence base into sustained, equitable access to MTMs. By situating MTMs within federal programs, aligning benefits with clinical needs, and ensuring adequate awareness and uptake, policymakers can help unlock the full potential of this intervention to improve health outcomes, reduce costs, and advance health equity.

### 4.1. Policy Solutions to Leverage the Clinical and Cost Advantages of Medically Tailored Meals

#### 4.1.1. Medicare FFS Demonstration for MTM

Under a four-year model, which aligns with bipartisan bills under consideration in the House and Senate, hospitals and clinicians would prescribe home-delivered MTMs to targeted Medicare FFS populations. This model would align with bipartisan legislation introduced in the Senate [33] and similar bipartisan legislation passed out of the House Ways and Means Committee [34] in the 118th Congress and reintroduced in the 119th Congress through the Medically Tailored Home-Delivered Meals Demonstration Pilot Act [35]. Unlike the legislation under consideration in Congress that applies only to Medicare Part A providers, this recommended model would apply to Part A and Part B providers. Availability under both Part A and Part B would allow for greater coordination and improved clinical outcomes in ongoing care, while also allowing the Innovation Center to explore which provider settings may offer the most beneficial outcomes in terms of both health improvements and cost savings. Key elements would include:

- Eligible beneficiaries: Eligible beneficiaries would include those who: (1) have one or more chronic disease(s); (2) are post-discharge; (3) are at risk for entering an institutional care setting; and/or (4) have limitations in activities of daily living (ADL).
- Eligible providers: Eligible providers would include inpatient and outpatient hospitals, as well as clinical group practices that have performed well historically in Medicare quality programs.
- Quality measures: Quality measures would assess reductions in: (1) all-cause inpatient admissions; (2) all-cause inpatient readmissions; (3) ED visits; and (4) post-acute institutional care.
- Cost measures: A cost measure would assess the reduction in total cost-of-care.
- Adherence to national nutrition guidelines: Home-delivered MTMs would have to adhere with the current and future Dietary Guidelines for Americans, which reflect standards for high-quality nutrition [36].
- No or minimal cost-sharing: Beneficiaries would not have any cost-sharing obligations for the home-delivered MTMs provided in the model or, in the alternative, may have modest cost sharing obligations, in order to encourage the widest possible participation to ensure a true test of the model.
- Awareness: By launching the program with providers instead of managed care plans, awareness among providers would be necessary, which would lead to greater member awareness. In addition to training, availability of information about MTM programs in provider electronic health records would further facilitate awareness of and timely discussions about, and patient referrals into, available MTM programs.

#### 4.1.2. Medicare Advantage High-Value FIM Benefit Model

Under the multi-year model, MA plans would be able to provide high-value home-delivered MTMs or other evidence-based FIM interventions to targeted MA populations, such as duals and those living with chronic disease. Beneficiaries would actively engage with their providers to choose home-delivered MTMs or the combinations of solutions that best meet their individual health needs to advance their overall health and well-being at a lower cost to Medicare. The inclusion of healthcare providers in the program could drive higher utilization of MTMs than is seen in MA supplemental benefits programs today by overcoming the member awareness barrier. Key elements of the model would include:

- High-value benefit design: MA plans would work with contracted food providers to offer home-delivered MTMs and/or other FIM solutions that adhere to national nutrition standards (the current and future Dietary Guidelines for Americans) to at-risk MA enrollee populations at little or no cost at durations that extend beyond the current MA supplemental or Special Supplemental Benefits for the Chronically Ill limitations.
- Eligible beneficiaries: Beneficiaries electing to receive FIM benefit could include those who: (1) have one or more chronic disease(s); (2) are dual-eligible; (3) are post-discharge; (4) are at risk for entering an institutional care setting; and/or (5) have ADL limitations.
- Value assessment: MA plans and Medicare would share in savings from reductions in total cost-of-care spending. Quality measures would assess reductions in: (1) all-cause inpatient admissions; (2) all-cause inpatient readmissions; (3) ED visits; and (4) post-acute institutional care.

#### 4.1.3. Use of FIM Benefits in Current CMMI Models

Certain models currently overseen by the Innovation Center allow for the provision of home-delivered MTMs—but not all participating entities (e.g., health systems, accountable care organizations, or state Medicaid agencies) may make the benefit available. Given the clinical and cost-effective value of home-delivered MTMs, the Innovation Center potentially could encourage those participants who have not yet made MTMs available to do so in the following models:

- Transforming Maternal Health (TMaH) Model: TMaH supports state Medicaid agencies in delivering whole-person maternity care by addressing physical, mental, and social needs during pregnancy. Participating states could incorporate home-delivered MTMs as part of care plans to improve maternal and infant health outcomes.
- Medicare Shared Savings Program (MSSP): Under the MSSP, Accountable Care Organizations (ACOs) can provide in-kind services such as meal programs to improve patient outcomes as part of broader care coordination to effectively manage chronic disease.
- ACO Realizing Equity, Access, and Community Health (REACH) Model: In ACO REACH, providers can offer home-delivered MTMs as in-kind service to advance patient care.
- Transforming Episode Accountability Model (TEAM): Under TEAM, acute care hospitals have responsibility for cost and quality of care for 30 days following a Medicare FFS patient undergoing one of five types of surgical procedures. All TEAM-participating hospitals could ensure that post-discharge patients have access to home-delivered MTMs to improve health outcomes and lower total costs.
- Medicare Diabetes Prevention Program (MDPP) Expanded Model: The MDPP gives beneficiaries with prediabetes access to structured individual and group interventions with the goal of preventing the onset of type 2 diabetes. Providing home-delivered MTMs would complement the overall strategy of the model to engage the at-risk beneficiary in better managing his health to prevent type 2 diabetes onset.
- Enhancing Oncology Model (EOM): EOM providers can offer home-delivered MTMs as in-kind services to improve health outcomes and the overall care experience for beneficiaries with cancer participating in the model.

While this narrative review highlights the clinical, observational, and modeled evidence for chronic care, post-discharge, and long-term care MTMs programs, and policy opportunities surrounding MTMs, several limitations should be acknowledged. First, as a narrative review, this analysis did not employ a systematic methodology and therefore may be subject to selection bias in the studies and reports included. The evidence base itself is heterogeneous, with variations in study design, populations served, intervention protocols, and outcome measures, which limits the ability to make direct comparisons across programs. Much of the data comes from case studies, evaluations by health plans, and grey literature, which, while valuable for understanding real-world implementation, may lack the rigor and generalizability of randomized controlled trials. Additionally, many interventions combined MTMs with other supports, such as counseling from an RD, nurse case management, or supplemental food resources, making it difficult to isolate the independent contribution of MTMs to observed outcomes. Most published evaluations are also short term, and few address sustainability, scalability, or long-term health and cost outcomes. Finally, the focus on U.S.-based studies means the findings may not be generalizable to other health systems. These limitations underscore the need for additional high-quality, longitudinal, and comparative research to strengthen the evidence base and further inform federal policy decisions.

## 5. Conclusions

This narrative review suggests that MTMs are a cost-effective and common-sense solution to America’s chronic disease crisis. They alleviate food and nutrition insecurity, assist in managing the leading causes of disease and death in the United States, and are associated with savings in health care. They also provide social benefit to both patients and caregivers as they seek to remain healthy and active in community settings, ultimately playing a foundational role as part of a broader FIM ecosystem. Policymakers have a unique opportunity to expand access to MTMs through programs such as a Medicare FFS demonstration. Health care leaders have an opportunity to ensure MTMs are incorporated into benefit packages and care plans, and that members, patients, and providers have a clear understanding of available MTM benefits and programs, and the ability to easily access those services. The success of these initiatives will require coordinated effort from all health care stakeholders to make scaling FIM successful and realize the potential cost savings of MTMs and other FIM solutions.

## Figures and Tables

**Table 1:** Summary of clinical, observational, and modeled evidence for chronic care, post-discharge, and long-term care medically tailored meals programs.

Program Type	Study Design	Population	Sample Size	Meals Protocol	Duration	Selected Outcomes	Reference
Chronic Care	Observational cohort	Dually eligible Medicare-Medicaid adults	133 medically tailored meals recipients with 1002 controls; 624 non-tailored with 1318 controls	Medically tailored meals: 2 meals + snacks/day, 5 days/week; Non-tailored: 2 meals/day, 5 days/week	6 months	Medically tailored meals: 70% fewer emergency department visits, 52% fewer hospital admissions, 72% fewer emergency transports, net savings $220/month; Non-tailored: 44% fewer emergency department visits, 38% fewer emergency transports, no change in hospital admissions, net savings $10/month	[11]
Chronic Care	Retrospective cohort	State of Massachusetts citizens with chronic conditions and data in the Massachusetts All-Payer Claims database	499 medically tailored meals recipients, 521 nonrecipients	10 medically tailored meals/week (2/day, 5 days/week), tailored to medical needs	Average of 12 months (median 9 month)	49% fewer hospital admissions (incidence rate ratio 0.51); 72% fewer skilled nursing admissions (incidence rate ratio 0.28); $753 lower monthly spending (16% reduction)	[9]
Chronic Care	Quasi-experimental	Elderly adults with diabetes in New York State	79 meal recipients; 75 controls	Home-delivered meals daily (3/day, 7 days/week)	Ongoing	Recipients had lower food insecurity (*p* < 0.02), better dietary adherence, less uncontrolled diabetes (22% vs. 52%, *p* = 0.03), and fewer hospitalizations for uncontrolled diabetes (7% vs. 23%, *p* = 0.09)	[12]
Chronic Care	Pilot cohort	Chronically ill adults with HIV/AIDS, cancer, renal disease, etc., in Philadelphia	65 Metropolitan Area Neighborhood Nutrition Alliance clients; 633 controls	3 tailored meals/day, 7 days/week, with nutrition counseling	≥3 months; outcomes tracked over 12 months	Lower monthly costs ($28,268 vs. $40,906, *p* < 0.001); 37% shorter hospital stays (10.7 vs. 17.1 days); fewer inpatient visits (0.2 vs. 0.4, *p* < 0.001); 93% discharged home vs. 72% (*p* < 0.001)	[13]
Chronic Care	Policy simulation model	U.S. adults with Medicare, Medicaid, or private insurance, diet-sensitive condition + instrumental activity of daily living limitation	6.3 million modeled eligible adults	10 tailored meals/week (2/day, 5 days/week)	8 months/year	1 year: 1.59 million fewer hospitalizations, $13.6 billion net savings; 10 years: 18.3 million fewer hospitalizations, $185.1 billion net savings	[14]
Chronic Care	Policy simulation model	Adults with Medicare, Medicaid, or private insurance; diet-sensitive condition + instrumental activity of daily living limitation	10.4 million modeled eligible adults	10 tailored meals/week (2/day, 5 days/week)	8 months/year	1 year: 2.6 million fewer hospitalizations, $23.7 billion savings; cost-saving in 49/50 states; 5 years: 10.8 million fewer hospitalizations, $111 billion in savings	[15]
Chronic Care	Single-arm feasibility cohort	Patients with chronic conditions	60 participants analyzed	14 frozen meals/week (2/day, 7 days/week)	3 months	Decreased emergency department visits (1.7 → 1.2, *p* = 0.005); decreased inpatient days (5.1 → 3.2, *p* = 0.02); average cost savings $12,046; high satisfaction; no change in mental/physical health scores	[16]
Chronic Care	Prospective pre-post cohort	Adults with HIV and/or type 2 diabetes, low-income, food insecure	72 participants; 52 completed with data	3 medically tailored meals + snacks/day, 7 days/week, meeting 100% of daily nutritional needs	6 months	Improved food security (very low: 60% → 12%); improve diet quality (reduced fat, improved fruits/veg); reduced depressive symptoms and binge drinking; reduced trade-offs between food and healthcare; HIV: improved antiretroviral therapy adherence (47% → 70%); Type 2 diabetes: reduced distress, improved self-management; HbA1c improved (not significant)	[17]
Chronic Care	Randomized cross-over trial	Adults with poorly controlled type 2 diabetes and food insecurity	44 participants randomized; 42 completed	10 tailored meals/week (2/day, 5 days/week), tailored for diabetes + comorbidities	12 weeks with meals, 12 weeks without (cross-over)	Increased diet quality (healthy eating index + 31 points, *p* < 0.0001); decreased food insecurity (42% vs. 62%); decreased hypoglycemia (47% vs. 64%); increased mental health quality of life; HbA1c modestly lower (not significant)	[18]
Chronic Care	Randomized controlled trial	Adults ≥ 60 years with hypertension and/or hyperlipidemia	210 participants analyzed (321 enrolled)	7 Dietary Approaches to Stop Hypertension meals/week (1/day), delivered frozen with dietitian follow-up	12 months	Increased intermediate Dietary Approaches to Stop Hypertension accordance (20% higher at 6 months, *p* = 0.001); increased saturated fat target compliance at 12 months (18% higher, *p* = 0.007); increased targets met for protein, fat, magnesium, potassium; gains stronger in whites and higher-income participants	[19]
Chronic Care	Prospective intervention trial	Adults with congestive heart disease	35 participants analyzed	3 heart-healthy meals + snacks daily (67% carb, 16% protein, 17% fat, 4% sat fat, 25 g fiber) + weekly dietitian phone education	8 weeks	91% adherence at 4 weeks, 88% at 8 weeks; decreased weight (−3.7 kg), decreased waist circumference (−2.0 inches), decreased LDL (−7.5 mg/dL), decreased total cholesterol (−7 mg/dL); improved diet quality and quality of life	[20]
Chronic Care	Prospective single arm trial	Adults with type 2 diabetes enrolled in a digital care program	Intervention: 154 enrolled (110 analyzed); Control: 203 (100 analyzed)	3 meals/day, 7 days/week (pre-portioned diabetes-friendly meals; household member option)	4 weeks	Time in range +6.8 points (56.1 → 62.9%; *p* < 0.001); estimated HbA1c −0.21 (*p* < 0.001); time above range −6.8 (*p* < 0.001); Difference-in-differences vs. control: +6.5, −0.18, −6.4, respectively; more participants achieved or maintained ≥70% time in range; effects diminished after meals ended	[21]
Chronic Care	Pilot prospective trial	Adults on maintenance hemodialysis	20 participants analyzed	3 meals lower in sodium, phosphorous and potassium	4 weeks (after 4-week control period)	Reduction in interdialytic weight gain (−0.82 kg, *p* < 0.001); reduce sodium intake (−1687 mg, *p* < 0.001); reduce thirst, xerostomia; reduce systolic blood pressure (−18 mmHg, *p* < 0.001) and diastolic blood pressure (−6 mmHg, *p* = 0.008); reduce plasma phosphorus; reduce volume overload; no change in serum/tissue sodium	[22]
Chronic Care	Randomized controlled trial	Adults with cirrhosis and symptomatic ascites	40 randomized, 20 received medically tailored meals, 20 received standard of care (low-sodium diet education handout)	3 low-sodium, high-protein meals/day + evening protein supplement	4 weeks meals, 12 weeks follow-up	Reduction in paracenteses (0.34 vs. 0.45/week); intervention group had fewer hospital days (0.62 vs. 1.04/week), lower diuretic escalation; increased ascites-specific quality of life (+25% vs. +13%); deaths: 2 vs. 4; 1 transplant in intervention arm	[23]
Post-Discharge	Comparative cohort	Medicare Advantage members ≥65, post-hospitalization for heart failure or other acute conditions	Heart failure: 742 meals vs. 3289 controls; Non-heart failure: 756 meals vs. 7188 controls	2–3 home-delivered meals/day, 7 days/week	Up to 4 weeks (56–84 meals)	Reduced 30-day mortality (odds ratio 0.37) and reduced composite rehospitalization + death vs. concurrent controls (odds ratio 0.55, *p* < 0.001). Non-heart failure: reduced 30-day mortality (odds ratio 0.26) and reduced composite events (odds ratio 0.48, *p* < 0.001); effects persisted at 60 days; time-to-readmission also improved (hazard ratio ~0.7)	[24]
Post-Discharge	Randomized controlled trial	Adults ≥ 55 discharged after acute decompensated heart failure	66 randomized, 33 received Dietary Approaches to Stop Hypertension/Sodium-Reduced Diet, 33 received usual care	3 Dietary Approaches to Stop Hypertension/Sodium-Reduced Diet meals/day + snacks, ~1500 mg sodium/day, tailored for comorbidities	4 weeks meals, 12 weeks follow-up	Kansas City Cardiomyopathy Questionnaire summary (+13 vs. +10, *p* = 0.37); Kansas City Cardiomyopathy Questionnaire clinical trended better (+18 vs. +10, *p* = 0.053); 30-d heart failure rehospitalizations lower (3 vs. 11, *p* = 0.055); fewer days rehospitalized (17 vs. 55, *p* = 0.06); no major safety concerns	[25]
Post-Discharge	Time-series evaluation within care transition program	Medicare members designated as at high risk of readmission and part of a care transitions program	622 Simply Delivered for Maine recipients	Weekly frozen specialized meals; up to 7 days/week supplied; first delivery ≤4 days post-discharge	4 weeks post-discharge (within the care-transition period)	30-day readmissions 10.3% (8.1–12.9); 16.3% lower vs. Community-based Care Transition Program-only 12.3% (10.0–15.2) and 38% lower vs. baseline 16.6% (13.8–19.7); estimated savings $212,160; program cost $43,540; return on investment 387%	[26]
Long-term Care	Policy simulation model	Adults ≥ 65 in Older Americans Act Title III-C2 programs (Meals on Wheels)	National model: ~393,000 added clients	Typically 1 meal/day, 5–7 days/week	Ongoing community support; model projects +1% expansion	1% expansion → 0.2% reduced low-care nursing home residents; ~$109 million Medicaid savings (1722 avoided nursing home placements); 26 states saved (e.g., PA $5.7 million), 22 states incurred net costs; meal expansion cost $117.6 million	[27]

**Table 2:** Medically tailored meals benefit availability gaps.

	Access Gaps
Medicare Fee-for Service	Medically tailored meals not covered as a Medicare Fee-for-Service benefit No supplemental benefits in Medicare Fee-for-Service
Medicare Advantage	Approximately 83% of Medicare Advantage beneficiaries have access to medically tailored meals supplemental benefits Benefits are mostly limited to short duration post-discharge programs and utilization is unclear [29]
Medicaid Home and Community-Based Waivers	Not all waivers include medically tailored meals Meal utilization among waiver-eligible limited; care attendants time misaligned [27,30,31]
Medicaid (other)	Novel coverage pathways (Value-Added Benefit, In Lieu of Services and Settings, 1115 Waivers) are fragmented States require specialized knowledge, budget, review to implement novel waivers

## Data Availability

No new data were created or analyzed in this study. The data supporting this narrative review’s results were obtained from PubMed and Google Scholar and are available in the supplied Table 1.

## Abbreviations

The following abbreviations are used in this manuscript:

FFS	Fee-for-service
MTM	Medically tailored meal
MA	Medicare Advantage
FIM	Food-is-medicine
CMMI	Center for Medicare and Medicaid Innovation
RD	Registered dietitian
ED	Emergency department
ADL	Activities of daily living
MSSP	Medicare shared savings program
ACO	Accountable Care Organizations
REACH	Realizing Equity, Access, and Community Health Model
TEAM	Transforming Episode Accountability Model
MDPP	Medicare Diabetes Prevention Program Expanded Model
EOM	Enhancing Oncology Model
TMaH	Transforming Maternal Health Model
